# Are hemipenial traits under sexual selection in *Tropidurus* lizards? Hemipenial development, male and female genital morphology, allometry and coevolution in *Tropidurus torquatus* (Squamata: Tropiduridae)

**DOI:** 10.1371/journal.pone.0219053

**Published:** 2019-07-10

**Authors:** Anderson Kennedy Soares De-Lima, Ingrid Pinheiro Paschoaletto, Lorena de Oliveira Pinho, Piktor Benmamman, Julia Klaczko

**Affiliations:** 1 Laboratory of Comparative Vertebrate Anatomy; Department of Physiological Sciences, University of Brasilia, Brasília, Federal District, Brazil; 2 Graduate Program of Zoology; Institute of Biological Sciences, University of Brasilia, Brasilia, Federal District, Brazil; University of Regina, CANADA

## Abstract

Male genitalia show considerable morphological variation among animals with internal fertilization and exhibit a high level of evolvability in lizards. Studies have suggested that sexual selection may be driving hemipenial evolution against natural selection and pleiotropy. Given the direct interaction of male and female genitals, coevolution of the aforementioned is posited by several hypotheses of genital evolution. However, there are only a few studies on female genitalia morphology, resulting in a lack of coevolution description and understanding. Studies of allometric patterns have filled some gaps by answering questions about male genital evolution and could prove a powerful tool in clarifying coevolution between male and female genitals. Here, we studied the genital morphology of *Tropidurus torquatus*. This *Tropidurus* lizard species is an emerging Neotropical lizard model organism notable for having enlarged hemipenial lobes in contrast with other tropidurid species. In this study, we analyzed hemipenial development in early and late stages, describing both morphological variation and ontogenetic allometric pattern. We used quantitative traits to describe male and female genital morphology, examining their static allometric patterns and correspondence. Our study provides a quantitative discussion on the evolution of lizard genitals, suggesting that sexual selection plays an important role in genital evolution in *Tropidurus* lizards.

## Introduction

Snake and lizard species (Squamata) possess a pair of intromittent male reproductive organs, called hemipenes. These organs are known to show considerable variation in shape, dimensions, ornamentation, and are often more divergent than non-genital traits [1–4]. It is common that closely related species tend to differ in hemipenial morphology, leading to the use of hemipenis as a key taxonomic and phylogenetic character [5–8]. In addition, the size of hemipenial traits has a higher evolutionary rate than other morphological traits [9,10]. Few studies investigating the ontogenetic development of hemipenial morphology exist, the majority of which focus on the first stages of development [11–13]. Therefore, little is known about how hemipenis morphological complexity and ornamentation are driven by developmental processes [11].

Several studies have suggested that sexual selection may be the main driver of hemipenial evolution. However, a recent study suggested that hemipenial morphological variation could be the result of pleiotropy and sexual selection acting simultaneously as antagonistic forces, in which the former would constrain evolutionary speed, while the latter would accelerate evolutionary rates [3,14–16]. Studies of allometric patterns, both static and ontogenetic, that have been extensively used to describe morphological variation in genitalia across species of insects and arthropods are still incipient for vertebrates, especially for squamates [17]. The allometric pattern is the description of the proportional size of a particular structure in relation to body size, calculated across multiple individuals in a population [18]. If the allometric pattern is measured across the developmental trajectory, it is called ontogenetic allometry [19]. Positive allometry has often been associated with traits under sexual selection. However, most of these conclusions were drawn exclusively from species with unusually expressed traits [17–21]. Nevertheless, Bonduriansky [22] showed that positive allometry in specific traits is not always a predictor of sexual selection. Eberhard [18,23] described the allometric patterns for male genitalia across several species with internal fertilization. He showed that the vast majority of the analyzed species have negative allometric slopes in their genitalia. Based on these results, he formulated the “one-size-fits-all” hypothesis, that is interpreted as evidence of stabilizing sexual selection, keeping male genital size constant in a way to match the average female genital size in the population [17,18,23].

The direct interaction between male and female genitals in animals with internal fertilization implies that their morphology would be coevolving, either by females selecting males as better stimulators or by an arms race between the sexes over the control of insemination and fertilization [24]. However, there is a lack of information about female genitalia morphology and consequently the role that coevolution plays in genital evolution [25]. Female Squamata genitalia is an internal organ which is soft and therefore challenging to study [24,26]. Few studies are available, however the majority of them focus on qualitative description of morphological variation, while relatively few compare morphologies between the sexes [24].

*Tropidurus torquatus* is an emerging Neotropical lizard model species which is widely distributed throughout South America and extremely abundant in open formations, especially in disturbed environments [27,28]. This species has been extensively studied in terms of ecology, phylogeny, physiology, and is one of the few South American lizards species to have a complete staging table of post-ovipositional development [29]. Here we investigate the hemipenial development and morphology of this organ in *T*. *torquatus*, together with an examination of the ontogenetic and static allometric pattern. We also described the female genital morphology of this species and analyzed its static allometric pattern. Finally, we compared the allometric slopes of hemipenial and female genital traits.

## Material and methods

### Embryo collection and processing

Ten gravid *Tropidurus torquatus* females were captured using the noose technique in urban areas of Brasilia (15°45’46.79”S 47°52’05.34”W), Federal District, Brazil, during the breeding season (Oct/2016 –Feb/2017). The specimens were kept in terrariums with medium grain vermiculite as a substrate until egg laying. In order to provide stable maintenance and minimize distress, four to five females were placed in 60 X 40 X 50 cm terrariums equipped with heating plates at 35°C for thermoregulation. Lizard alimentation consisted of live cockroaches (*Nauphoetacinerea*) floured with calcium and vitamin supplements for reptiles, with fresh water *ad libitum*. The room was maintained at ambient temperature (~25°C) with a daylight cycle of 12h.

Terrariums were inspected daily for the presence of eggs. Once spawning occurred, clutches were individually kept in 50ml recipients containing 10mg of vermiculite and 20ml of water, conferring a humidity of 100%. The recipients were placed in an egg incubator with a constant temperature of 30°C. Eggs were dissected daily throughout the incubation period in an isotonic saline solution (0.75% NaCl). In total, two to seven embryos were analyzed per stage giving a final sample of 57 embryos (Please refer to Table A in S1 File for a detailed specimen list). Immediate embryo euthanasia was performed using 5ml of 2% lidocaine hydrochloride dissolved in the saline solution during dissection. On completion of *in vivo* photographic documentation, the embryos were cold fixed in Carson fixative solution for 24-48h and stored in 70% ethanol. All captures were licensed by ICMBio/IBAMA under permit n° 55406–1. This study was approved by the University of Brasilia Ethics Committee for Animal Use (UnBDOC n° 166980/2013).

Fifteen embryos were submitted to Scanning Electron Microscopy in order to have a detailed visualization of the hemipenial morphological changes during embryonic development at different embryonic stages. Samples that had been previously cold-fixed in Karnovsky Fixative for 24 hours were: immersed in a solution of 2% osmium tetroxide, dehydrated in crescent solutions of acetone, critical-point dried with CO_2_ in a Balzers CPD030 and coated with gold in a Leica EM SCD005 Sputter Coater. Samples were analysed using a JEOL JSM7000F Scanning Electron Microscope.

General developmental modifications of genitalia were described stage-by-stage, based on the embryonic staging table of *Tropidurus torquatus* [29] and the vertebrate staging system [30].

## Adult genitalia preparation and description

A description of genital morphology and an intraspecific comparison were made using both hemipenes from 20 specimens of *Tropidurus torquatus*, thus totaling 40 hemipenes, together with the female internal genitalia of 20 females (detailed specimen lists are provided in the Supplementary Information section, Tables C and D in S1 Table). All of the specimens were collected in urban areas of Brasilia, Distrito Federal, Brazil (ICMBio/IBAMA under permits n° 55406–1). The specimens were euthanized by intraperitoneal administration of lidocaine hydrochloride (1.5ml at 2% concentration) in accordance with the methodology guide of the University of Brasilia Ethics Committee (CEUA-UnB).

For the male specimens that did not everted hemipenes during the fixative procedure, we followed the Pesantes method [31]. A small incision was made at the base of the tail. Both hemipenes were removed, immersed in 1.5% potassium hydroxide solution for up to three minutes and manually everted using forceps. After eversion, the hemipenes were washed in a 70% ethanol solution to dilute any remaining potassium hydroxide. Finally, red petroleum jelly was injected to facilitate ornamentation visualization and a knot was made at the hemipenial base using surgical thread.

Female internal genitalia were obtained by dissection under stereomicroscope of previously formaldehyde-fixed female specimens deposited in the LACV Scientific Collection. The cloaca was accessed by exposing the urogenital/digestive tract with a ventral incision in the abdominal region. Once exposed, the cloaca was extracted by dissection of the surrounding external face of the cloacal lips. Finally, the cloaca was transferred to a Petri dish and dissected along the coronal section plan to exhibit the ventral and dorsal internal faces.

Hemipenial descriptions were made using Klever & Bohme [32] and Dowling & Savage [2] terminology, with female cloacal terminology in accordance with Sánchez-Martinez *et al*. [33].

The specimens and corresponding genitals used in this study were deposited in the Laboratory of Comparative Vertebrate Anatomy Scientific Collection, Department of Physiological Sciences, Institute of Biological Sciences, University of Brasilia (LACV/ CFS/IB/UnB). Please refer to the Supplementary Material for a complete list of the specimens analyzed.

### Morphological measurements and statistical analysis

Hemipenes of embryos (n = 19) and adults (n = 40), and internal female genitalia (n = 20) were photographed under a Nikon SZM460 stereomicroscope coupled with a Cannon Power Shot digital camera. Morphometric measurements were recorded for the following traits: hemipenial truncus length (TCL), hemipenial truncus width (TW), hemipenial lobe length (LL) and hemipenial total length (TTL). The following traits were measured for female cloaca: proctodeal-urodeal region length (FPUR), urodeal corn length (FUC), and female cloacal total length (FIGL). All of the measurements were taken using the ImageJ software. Measurements of the snout-vent length (SVL) were also taken using a caliper rule as a proxy for body size for embryo and adult specimens (raw data are provided in the Supplementary Information section, Tables B, C and D in S1 Table).

Asymmetry between left and right hemipenial traits was tested for adult male hemipenes using Paired *t*-tests. Ontogenetic and static allometry was also described for embryonic and adult hemipenes, in addition to the aforementioned cloacal traits. The allometric coefficient (*b*) was estimated as the slope of a linear regression of trait values against SVL [18]. Each trait was analyzed to determine whether the allometric coefficient was significantly different from the isometry (*b* = 1) using the slope test function on Smatr R software package [34,35]. Coefficient values higher than one indicate positive allometry, while a value lower than one indicates negative allometry.

Finally, we tested the morphological correspondence of genitalia among sexes using ANCOVA tests for differences between male-female linear regressions. Differences in allometric coefficients (slope) and male-female body size (intercept) were investigated for each male-female trait: TCL vs. FPUR, LL vs. FUC, and TTL vs. FIGL. All analysis and graphics were performed using the R software [34,35].

## Results

### Early development of cloaca and genital primordium

**Fig 1:**
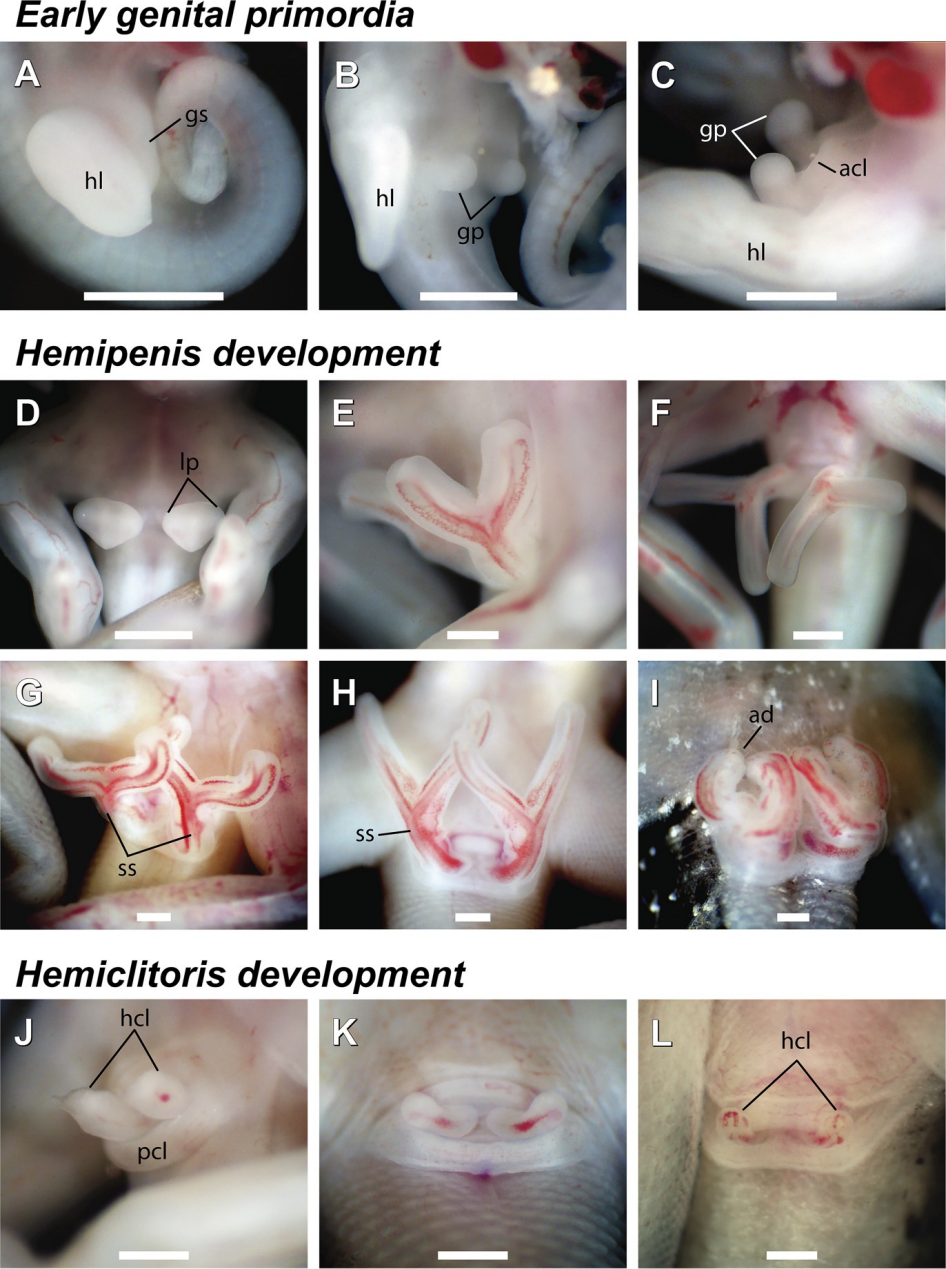
Genital development of *Tropidurus torquatus*. A, stage 29; B, stage 33; C, stage 34; D; stage 35; E, early stage 38; F, late stage 38; G, early stage 39; H, late stage 39; I, stage 40; J, late stage 37; K, stage 39; L, stage 40. Legends: acl, anterior cloacal lip; ad, apical disc; gp, genital primordia; gs, genital swelling; hcl, hemiclitoris; hl, hind limb; lp, lobe primordia; pcl, posterior cloacal lip; ss, spermatic sulcus. Scale bar: 0.5mm.

**Fig 2:**
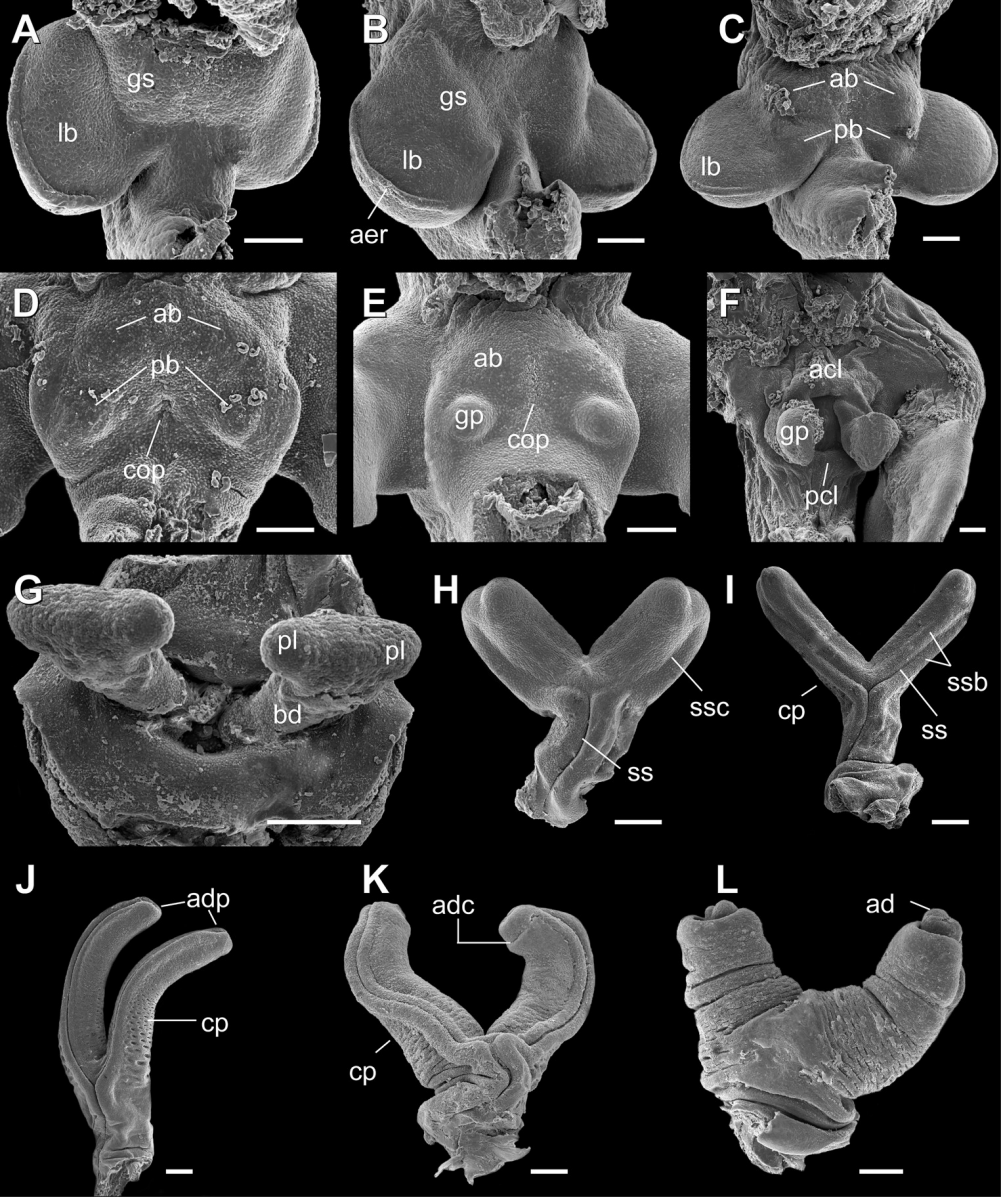
Hemipenial development of *Tropidurus torquatus* under SEM analysis. A, stage 28; B, stage 29; C; stage 30; D, early stage 31; E, late stage 31; F, late stage 32; G, stage 36; H, stage 37; I, stage 38; J, stage 40; K, stage 41; L, stage 42. Legends: ab, anterior bud; acl, anterior cloacal lip; ad, apical disc; adc, apical disc constriction; adp, apical disc primordia; aer, apical epidermal ridge; tr, hemipenial truncus; cop, cloacal opening primordia; cp, calyces primordia; gp, genital primordia; gs, genital swelling; lb, hind limb bud.; pb, posterior bud; pcl, posterior cloacal lip; pl, primordium of lobes; ss, spermatic sulcus; ssb, spermatic sulcus borders; ssc, spermatic sulcus constriction. Scale bar: A-F, 100μm; G-L, 200μm.

Initial development of the genital-cloacal morphology occurs relatively quickly in the early post ovipositional stages. External genitalia development begins two days postoviposition (DPO), at the ovipositional stage (Stage 28). At this stage, presence of a genital swelling at the ventral-proximal portion of the hind limb buds is distinguishable(Fig 1A and Fig 2A and 2B). Two pairs of bulbs located ventromedially in the cloacal region arise from the genital swelling at the 6 DPO (Fig 2C). From stages 31 to 32 (8–11 and 11–14 DPO, respectively), remarkable modifications occur in the genital-cloacal morphology. At 9 DPO, a condensation of cells forms an epidermal depression along the region that further will develop into the cloacal opening (Fig 2D). By 10 DPO, the posterior bulbs develop into distinct genital primordia, whereas anterior bulbs further develop into the anterior cloacal lip, by 11 to 13 DPO, being totally fused by 14 DPO (Fig 2E and 2F).

### Late development of hemipenes

Late development of the hemipenial morphology is mainly characterized by increased length of the hemipenial truncus and lobes together with further emergence of ornamentation and apical disc in the latter stages. By stage 35, the apical region of genital primordia shows a laterally expanded aspect in male genitalia which further develops into two lobes. From this stage it is possible to distinguish between male and female embryos by the presence of this distal expansion (Fig 1D).

Apical expansion of the hemipenial truncus protrudes laterally by stage 36 giving origin to the hemipenial lobes, which undergo a rapid increase in length until stage 37 (Fig 2G and 2H). The “Y–shaped” hemipenes in stage 37 show a well-marked spermatic sulcus at the hemipenial truncus which is less marked along the lobes (Fig 2H). At stage 38, lobe length exceeds truncus length and the spermatic sulcus are now well developed along the lobes (Fig 2I). In the apical region, each lobe showed a marked constriction, that will give way to the apical disc margins in the following stage. The spermatic sulcus is fully formed along the lobes at stage 39, ending on the formed apical disc (Fig 1G and 1H and Fig 2J and 2K). At the end of stage 40, a great number of pits give rise to the primordium or ornamentation surrounding the lobes (Fig 2J). By the end of stage 41, the hemipenes start the inversion process to the inner side of the cloaca (Fig 1I and Fig 2L).

In females, modifications in the hemiclitoris are restricted to little increase in size during embryonic development from stage 35 to stage 41, whereby complete genital inversion occurs (Fig 1J and 1L).

### Adult genital morphology

**Fig 3:**
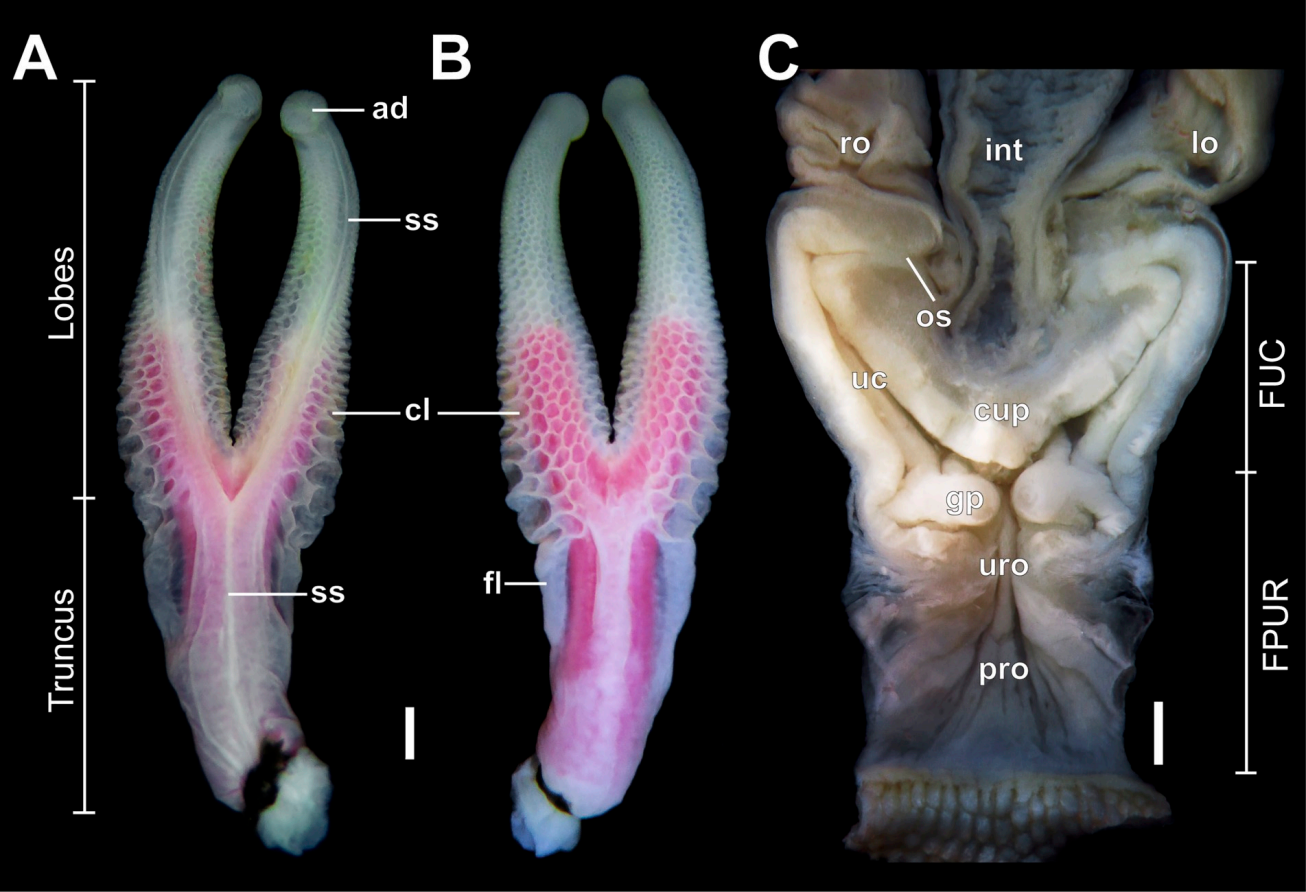
Male and female genital morphology of *Tropidurus torquatus*. Measurements are indicated for the hemipenial truncus, hemipenial lobes, female proctodeal-urodeal cloacal region (FPUR), and female urodeal corns (FUC). Right hemipenis in (A) ventral and (B) dorsal view, and female internal genitalia (C). Figure legends: ad, apical disc; gp, genital papilla; int, intestine; lo, left oviduct; os, oviduct sphincter; ro, right oviduct; uc, urodeal corn; uro, urodeal region; pro, proctodeal region; ss, spermatic sulcus. Scale bar: 1mm.

**Fig 4:**
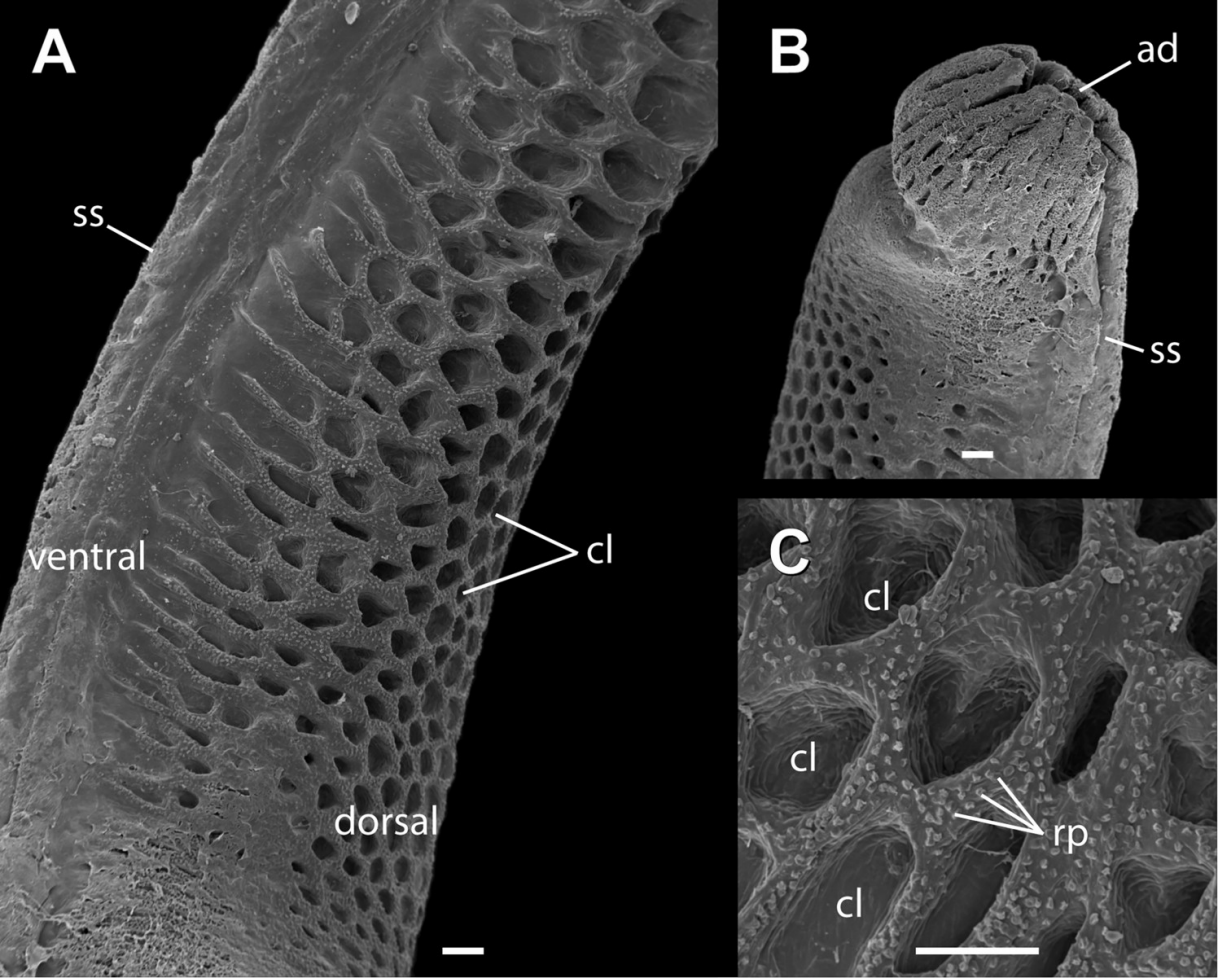
Adult hemipenis of *Tropidurus torquatus* under SEM analysis. (A) calyces, (B) apical disc and (C) reduced papilla. Figure legends: ad, apical disc; cl, calyces; rp, reduced papilla; ss, spermatic sulcus. Scale bar: 100μm.

*Tropidurus torquatus* adult males show deeply bilobed hemipenes, with lobe length measuring little more than truncus length (*t* = 3.797; *p* = 0.001) (Fig 3C; Table 1). The “Y-shaped” spermatic sulcus arises proximally, forking at the level of lobe bifurcation and ending distally at the apical disc. The hemipenial truncus is nude and slightly thicker than the hemipenial base. Both sulcate and asulcate faces of the truncus are nude, although marked laterally by the presence of a flounce that enlarges at the base of the lobes and is continuous with lobe ornamentation. The lobes are fully ornamented with calyces with the exception of the sulcate region. These calyces are deeply pronounced on the asulcate surface, decrease in size distally and are greatly reduced near the apical region (Fig 3A and 3B). The SEM analysis shows the presence of a great number of reduced papilla surrounding the margins of the calyces, distributed along the whole lobe. Additionally, characterization of the spermatic sulcus under SEM analysis shows it is continuous with the apical disc and the presence of a great number of parallel clefts that surround the apical disc (Fig 4). Finally, no statistical significance between right and left hemipenial traits was observed (Fig 3D–3G; Table 1).

**Table 1:** A comparison of right and left hemipenial trait values of *Tropidurus torquatus*.

Trait	Right HP	Left HP	*t*	*P*
TCL	6.04 ± 0.86	5.89 ± 0.71	0.5434	0.591
TW	2.59 ± 0.41	2.36 ± 0.36	1.7297	0.094
LL	7.90 ± 1.76	8.10 ± 1.44	-0.35245	0.727
TTL	13.94 ± 2.32	13.99 ± 1.85	-0.065255	0.948

Mean and Standard Deviation of: truncus length (TCL); truncus width (TW); lobe length (LL), and total length (TTL) in right and left hemipenes. Asymmetry results (*t*) and significance (*P*).

Female internal genital morphology is also marked by its “Y” shape. The cloacal lumen shows a folded mucosa, with the proximal region, proctodeal-urodeal (FPUR) measuring 4.85±1.03mm.The distal region, the urodeal corns (FUC), showed a length of 5.23±0.89mm, giving a total length (FIGL) of 10.08±1.72mm. The urodeal corns are deeply bilobed, comprising approximately the same length as the proctodeal-urodeal region (*t* = 1.244; *p* = 0.2213).

### Ontogenetic and static allometry: Male-female genital correspondence

**Fig 5:**
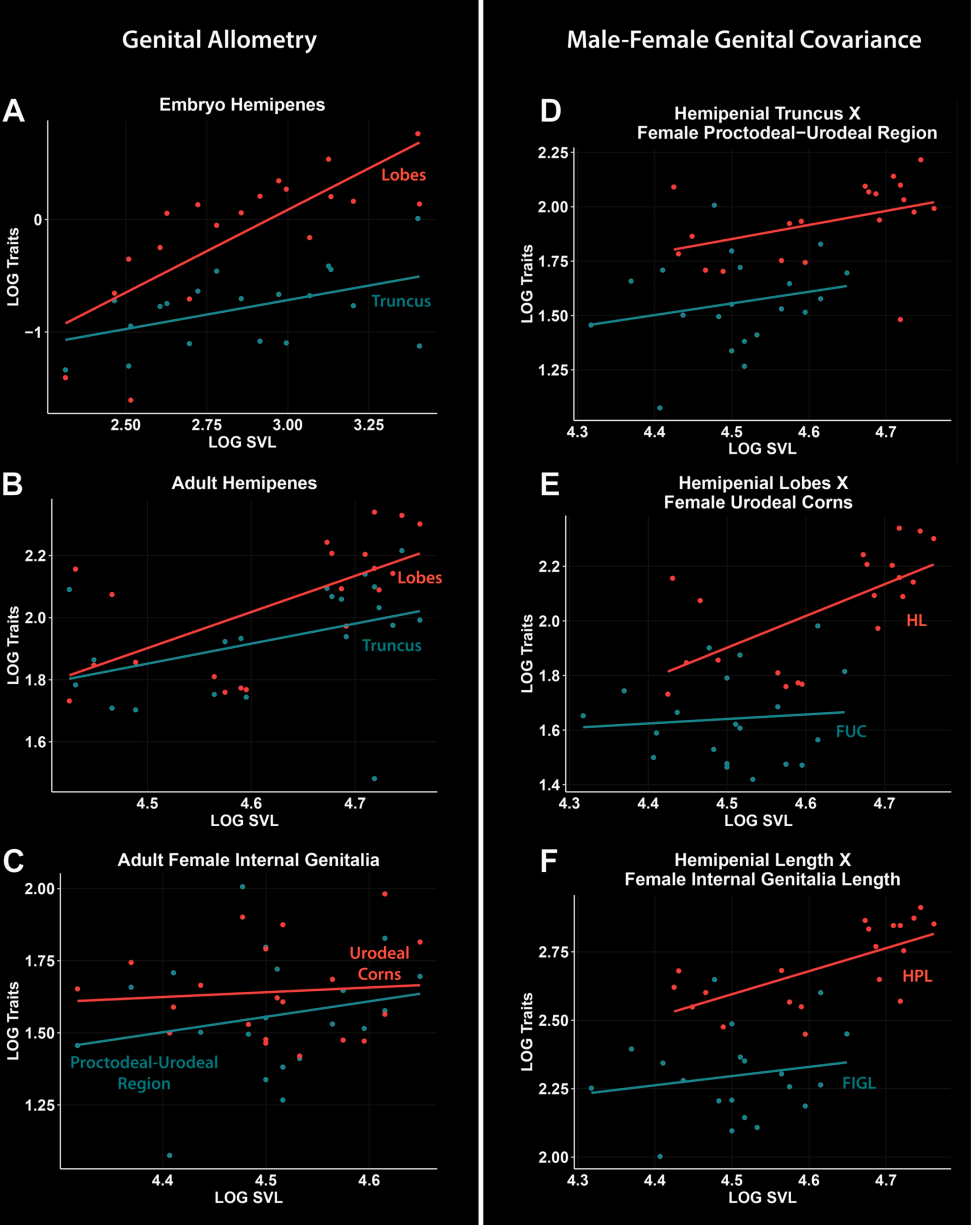
Regressions of log-transformed traits vs. log-transformed body size (SVL) for genital traits of *Tropidurus torquatus*. A-C: regression for hemipenial traits of embryos (A), hemipenial traits of adults (B), and female internal genitalia (C). D-F: comparison of male-female trait regressions for hemipenial truncus *vs* female proctodeal-urodeal region (D), hemipenial lobes *vs*. female urodeal corns (E), and hemipenial total length *vs*. female internal genitalia total length (F). Figure legends: FIGL, female internal genitalia total length; FUC, female urodeal corns; FPUR, female proctodeal-urodeal cloacal region; HL, hemipenial lobes; HPL, hemipenes total length; HT, hemipenial truncus.

For embryos, the allometric slope for truncus length was not significantly different from 1.0, indicating isometry, but slopes for lobe and total length were significantly greater than 1.0, thus suggesting positive allometry during ontogenetic development of these traits (Table 2; Fig 5A). For adults, allometric slopes for truncus length and total length were not significantly different from 1.0, tending, rather to a negative allometry for the total length. On the other hand, the slope for lobe length was significantly greater than 1.0, thus indicating positive allometry for this trait (Table 2; Fig 5B). All of the analyzed female genital traits showed negative allometry, with slopes significantly lower than 1.0.

**Table 2:** Summary of the statistics for ordinary least square (OLS) regression of traits *vs*. body size (SVL).

Embryo hemipenes (n = 19)	*b*	*r^2^*	*P*
Truncus length	0.514	0.232	0.7643
Lobe length	1.469	0.587	<0.001
Total length	1.093	0.605	0.0359
Adult male hemipenes (n = 20)	*b*	*r^2^*	*P*
Truncus length	0.645	0.162	0.035
Lobe length	1.164	0.415	0.002
Total length	0.841	0.442	0.195
Female cloaca (n = 20)	*b*	*r^2^*	*P*
Proctodeal-urodeal length	0.535	0.047	<0.001
Urodeal corn length	0.165	0.007	0.008
Total length	0.335	0.031	0.007

Allometric coefficient (*b*), coefficients of determination (*r^2^*) and significance deviation from a slope of one (*P*).

Male and female allometric patterns showed significantly different intercept values for all of the analyzed traits (Table 3). On the other hand, the slopes of the genital traits did not significantly differ (Table 3), although the hemipenial lobe length slopes were greater than the urodeal corn length slopes (Table 2).

**Table 3:** ANCOVA results for difference in genitalia between sexes. *F*-statistics, and *P*-values for each trait analyzed.

Trait	Slope		Intercept	
	*F*	*P*	*F*	*P*
Hemipenial truncus *vs*. female proctodeal-urodeal region	0.029	0.866	18.272	<0.001
Hemipenial lobes *vs*. female urodeal corns	3.278	0.079	23.970	<0.001
Hemipenial length *vs*. female internal genitalia length	1.148	0.291	38.231	<0.001

## Discussion

Male genitalia displays considerable morphological variation among animals with internal fertilization and exhibit high levels of evolvability in lizards [9]. In the last decades, biologists all over the world have been interested in explaining the developmental, genetic and evolutionary processes behind morphological diversity [36]. Although extremely diverse, the squamate clade is underrepresented with regards to ontogenetic studies, especially for soft tissue organs, such as hemipenes. Most of the hemipenial studies have focused on morphological and genetic processes exclusively during early developmental stages [13,37]. In this work, we investigate the genital morphology of the male Amazon lava lizard—*Tropidurus torquatus*—following a morphological and developmental approach, representing the first refined investigation for a tropidurid lizard. We have quantitatively described the morphology of male and female genitalia and demonstrated the correspondence between them. Finally, we showed that static and ontogenetic allometry along hemipenial traits in this species are the result of a rapid hemipenial growth during the late embryonic stages.

Among tropidurid lizards, morphological investigations of hemipenes are limited to its use in phylogenetic analysis [38,39]. Moreover, *Tropidurus* genus hemipenial morphology descriptions are scarce [40]. Even in phylogenetic analyses, hemipenial characteristics have been underestimated. Frost *et al*., [38,39] listed the general morphology of the genus *Tropidurus* with a combination of three characters: elongated hemipenial lobes, without apical disc and with calyces starting at the level of the lobes. Our data are in accordance with this description with regards to lobes and ornamentation. However, we note that *T*. *torquatus* (and some other species not formally included in this work, i.e. *T*. *oreadicus* and *T*. *hispidus*) have a distinct disc on the apical region of each lobe (Fig 4B). A possible explanation for these dissonant results could arise from the difficult reversibility of the apical region during hemipenial preparation of fixed specimens.

### Development

Recent work has demonstrated that hind limb and hemipenial buds share the same morphogenetic basis [41]. This recent finding has mitigated questions regarding the degree of pleiotropy in early developmental stages driving evolutionary modifications relating to hind limbs and hemipenes in lizards [3,15]. The development of the hemipenial buds and cloacal lips in *Tropidurus torquatus* is congruent with other Squamate species, showing an association between these structures in early developmental stages [13,29,37]. Similar to other recently investigated species, *Anolis carolinensis* and *Python regius*, our SEM analysis shows a close association between hind limb buds and genital swelling (Fig 2A–2F) [13,37].

The independent primordia for genital and anterior cloacal lip buds, first reported in the general embryonic staging table of *Tropidurus torquatus* [29], is now confirmed (Figs 1A–2C and Fig 2A–2E). Our results show that the genital swelling gives rise to genital and anterior cloacal buds, which follow independent developmental trajectories. Moreover, the development of genital primordia posteriorly to the anterior external cloacal buds occurs as in the other investigated squamates [13,37]. However, we did not exclude the possibility that the anterior cloacal buds may participate in genital bud formation in other squamate lineages due to the particular aspects of genetic regulation of hemipenial-cloacal development [13,41], and especially the possibility of migration of the genital bud in early embryogenesis [12]. Further developmental studies for underrepresented squamate lineages may clarify the degree of structural association during the development of the genital primordia and external cloacal structures.

Detailed information about late hemipenial development is even scarcer. Our SEM analysis reveals that all ornamental structures found in adult morphology begin their development during embryonic development, especially the primordia of the calyx and the apical disc structures (Fig 2J–2L). As such, post-embryonic development of hemipenes in *Tropidurus torquatus* may be restricted to its growth and remodeling of ornamental structures raised in late embryonic stages. A similar situation occurs in the gymnophthalmid lizard *Calyptommatus sinebrachiatus*, in which hemipenes show ornamental structures in late developmental stages, recognized even under stereoscopic analysis [42]. Once ornamental structures have been recognized as crucial for differentiation between cryptic species with hemipenial variation [5,6,43], late embryological studies may explain this variation and should be considered in studies of hemipenial evolution driven by processes such as natural and sexual selection [15,44].

### Is the allometric pattern of hemipenial lobes in *Tropidurus* lizards driven by male-female genital correspondence?

The size and shape of hemipenial lobes may itself be a source of general variation affecting genital morphology among Squamates [1,4,5,45]. The enlarged lobe condition is commonly found in different squamate groups [3]. Moreover, among tropidurid lizards, hemipenial lobes are short in all *Stenocercus* and long in all *Tropidurus* and other related genera [38,39].

This work presents the first study of ontogenetic hemipenial allometry among squamates and constitutes the second of static allometry in hemipenial traits. The static allometric pattern of anole lizards, a subspecies of *Anolis graham*, was recently published [14]. Similar to *A*. *grahami*, *Tropidurus torquatus* shows an isometric pattern for hemipenial total length, with a tendency towards negative allometry, and negative allometry for truncus length (Table 2). However, for *T*. *torquatus*, the allometric pattern for the hemipenial lobes is positive and is driven by ontogenetic allometry, whereby the lobes undergo rapid growth in late embryonic stages (Table 2).

Considering that allometric results of genital traits are a predictor of sexual selection [10,23,36,46,47], the allometric pattern found among *Tropidurus* hemipenial lobes could be explained by directional selection over this trait, caused as an action of two non-exclusive mechanisms: female cryptic choice and genital coevolution, as following discussed.

The direct interaction of genitalia during copula is directly related to reproductive success, especially when this process is led by female choice [24]. The general “female cryptic choice” hypothesis predicts that male genitalia has a stimulatory function and females choose to copulate with males whose hemipenial morphology better fulfils that stimulating function [47]. Likewise, a growing number of works have discussed genital coevolution under this hypothesis [24,25,48–51]. Morphological characterization of the vagina-cloacal region of *Tropidurus* lizards found a large number of epithelial glands surrounding the anterior part of the urodeal region [33]. So, if female cryptic choice is acting on *Tropidurus* genital evolution, the long hemipenial lobes may function as a stimulatory structure of female urodeal glands, constituting an indicator of good genes [52] or even serving as an efficient conductive system by which seminal fluid may reach the oviductal opening (Fig 3C).

Genital morphological interaction, be it congruent or antagonistic, is expected to reflect consequences on genitalia evolution, reproductive success, and speciation [24,51,53]. Moreover, the significance of studies of genital interaction during copula can be recognized from morphological, physiological, ecological and evolutionary perspectives [24,54]. Using artificial hemipenial eversion inside female cloaca and micro CT scanning in the garter snake *Thamnophis radix*, Brennan [24] showed that interaction of genitalia could occur even when unexpected. In this species, the hemipenial “T” shape turns to a “Y” when inflated inside the female cloaca, whereby the hemipenial lobes reach the vaginal pouch. As in *Tropidurus torquatus* (Fig 3), the degree of this morphological interaction may have a significant impact on insemination success from a stimulatory perspective.

Evidence for different tetrapod groups including lizards [55], demonstrates that the cloacal urodeal corns region has a phagocytic function with respect to spermatozoa, acting as a physiological barrier in sperm selection (for a brief review see Nogueira *et al*. [55].Therefore, the occurrence of phagocytic cells specifically on the cloacal urodeal corns further supports the need for evolutionary strategies that better fit this post-copulatory selective mechanism. This becomes especially true for species whose female oviductal opening is located distal to the urodeal corns, which is the case for the *Tropidurus* species studied (this study, Fig 3C; Sánchez-Martínez [33]), thus representing an additional source of selective pressure for hemipenial evolution [48]. More comparative cloacal studies are needed to further address this additional hypothesis of genital coevolution among lizards, as this study stands as the first quantitative evidence.

In reference to the demonstrated correspondence between the male and female genital morphology of *Tropidurus* lizards (Table 3; Fig 5D–5F), and assuming the possibility of hemipenial variation led by plasticity and ontogenetic effects [11,56,57], males with large lobes could benefit in both aspects. Firstly, the major stimulatory capability of the urodeal glands and, secondly by the amount of secretion produced by these glands which can cause a mechanical and/or biochemical difficulty for semen from future copulations. Evidence of female mate choice and sperm competition among lizards has been demonstrated in some case studies [10,58–60]. In the polymorphic phrynosomatid lizard *U*. *stansburiana*, it was is demonstrated that genital morphology is particularly associated with mating strategy, whereby “usurper male morphs” evolve a wider apical horn in contrast to other morphs [10]. To date, it has been demonstrated that for *T*. *torquatus*, male fitness plays a decisive role in the formation of harems [61]. However, experiments to better understand female choice and male territorial dominance are still lacking.

Despite the positive allometric pattern found in hemipenial lobes, the hemipenial total length in *Tropidurus torquatus* was determined as isometric, tending towards negative allometry (Table 2), supporting the “one-size-fits-all” theory [18,22]. This hypothesis predicts that males would be selected to have genitals that fit the average size of female genitals in the population, regardless of body size [18]. Thus, despite a possible directional selection in lobe size, the overall hemipenial length in *T*. *torquatus* could be destabilizing sexual selection, corroborating the finding in *Anolis grahami* populations [14]. Moreover, it is plausible that different factors such as pleiotropy, natural and sexual selection, and male-female genital coevolution could be antagonistically driving the evolution of particular traits of *Tropidurus* male genitalia as argued in recent studies [15,41,48,51,62].

## Supporting information

S1 Table **Table A. Embryonic analysis sample size and staging**. Number of analyzed embryos on stereoscopic microscopy (external morphology) and Scanning Electron Microscopy (SEM analysis) wit correspondent stages and days of post-oviposition (DPO).
**Table B. Morphometry of developing hemipenes**. Vouchers and measurements for the analyzed embryonic specimens: body size predictor (snout-vent length, SVL), hemipenial truncus length (TL), hemipenial lobes length (LL) and hemipenial total length (HTL). Values are given in millimeters.
**Table C (next page). Morphometry of adult hemipenes**. Vouchers and measurements for the analyzed adult male specimens: body size predictor (snout-vent length, SVL), hemipenial truncus length (TCL), hemipenial truncus width (TW), hemipenial lobes length (LL) and hemipenial total length (HTL). Values are given in millimeters.
**Table D. Morphometry of adult female cloaca**. Vouchers and measurements for the analyzed for the analyzed adult female specimens: body size predictor (SVL), proctodeal-urodeal region length (FPUR), urodeal corns length (FUC), and female cloacal total length (FCTL). Values are given in millimeters.
(DOCX)
