# Supplementary material for: Are hemipenial traits under sexual selection in Tropidurus lizards? Hemipenial development, male and female genital morphology, allometry and coevolution in Tropidurus torquatus (Squamata: Tropiduridae)
Source: PLoS One. 2019 Jul 10;14(7):e0219053. doi: 10.1371/journal.pone.0219053 (PMC6619691; doi:10.1371/journal.pone.0219053)
Supplement: S1 Table — Table A. Embryonic analysis sample size and staging. Number of analyzed embryos on stereoscopic microscopy (external morphology) and Scanning Electron Microscopy (SEM analysis) wit correspondent stages and days of post-oviposition (DPO). Table B. Morphometry of developing hemipenes. Vouchers and measurements for the analyzed embryonic specimens: body size predictor (snout-vent length, SVL), hemipenial truncus length (TL), hemipenial lobes length (LL) and hemipenial total length (HTL). Values are given in millimeters. Table C (next page). Morphometry of adult hemipenes. Vouchers and measurements for the analyzed adult male specimens: body size predictor (snout-vent length, SVL), hemipenial truncus length (TCL), hemipenial truncus width (TW), hemipenial lobes length (LL) and hemipenial total length (HTL). Values are given in millimeters. Table D. Morphometry of adult female cloaca. Vouchers and measurements for the analyzed for the analyzed adult female specimens: body size predictor (SVL), proctodeal-urodeal region length (FPUR), urodeal corns length (FUC), and female cloacal total length (FCTL). Values are given in millimeters. (DOCX) [file pone.0219053.s001.docx]

***PLoS One* Supporting Information Appendix S1**

Article title: Are hemipenial traits under sexual selection in Tropidurus lizards? Hemipenial development, male and female genital morphology, allometry and coevolution in Tropidurus torquatus (Squamata: Tropiduridae)

Authors: Anderson Kennedy Soares De-Lima, Ingrid Pinheiro Paschoaletto, Lorena de Oliveira Pinho, Piktor Benmamman & Julia Klaczko

The following Supporting Information is available for this article:

**S1 Table A. Embryonic analysis sample size and staging.** Number of analyzed embryos on stereoscopic microscopy (external morphology) and Scanning Electron Microscopy (SEM analysis) wit correspondent stages and days of post-oviposition (DPO).

**S1 Table B. Morphometry of developing hemipenes.** Vouchers and measurements for the analyzed embryonic specimens: body size predictor (snout-vent length, SVL), hemipenial truncus length (TL), hemipenial lobes length (LL) and hemipenial total length (HTL). Values are given in millimeters.

**S1 Table C (next page). Morphometry of adult hemipenes.** Vouchers and measurements for the analyzed adult male specimens: body size predictor (snout-vent length, SVL), hemipenial truncus length (TCL), hemipenial truncus width (TW), hemipenial lobes length (LL) and hemipenial total length (HTL). Values are given in millimeters.

**S1 Table D. Morphometry of adult female cloaca.** Vouchers and measurements for the analyzed for the analyzed adult female specimens: body size predictor (SVL), proctodeal-urodeal region length (FPUR), urodeal corns length (FUC), and female cloacal total length (FCTL). Values are given in millimeters.

**S1 Table A. Embryonic analysis sample size and staging.** Number of analyzed embryos on stereoscopic microscopy (external morphology) and Scanning Electron Microscopy (SEM analysis) wit correspondent stages and days of post-oviposition (DPO).

| Stage | DPO | N (external morphology) | N (SEM analysis) |
| --- | --- | --- | --- |
| 28 | 1-2 | 3 | 1 |
| 29 | 3-5 | 2 | 1 |
| 30 | 6-8 | 6 | 1 |
| 31 | 8-11 | 3 | 1 |
| 32 | 11-14 | 6 | 1 |
| 33 | 13-17 | 3 | 1 |
| 34 | 18-20 | 2 | 1 |
| 35 | 21-23 | 4 | 1 |
| 36 | 24-26 | 4 | 1 |
| 37 | 27-29 | 3 | 1 |
| 38 | 30-38 | 4 | 1 |
| 39 | 39-50 | 4 | 1 |
| 40 | 51-59 | 7 | 1 |
| 41 | 60-67 | 3 | 1 |
| 42 | 68-75 | 3 | 1 |
| TOTAL | 1 – 75 | 57 | 15 |

**S1 Table B. Morphometry of developing hemipenes.** Vouchers and measurements for the analyzed embryonic specimens: body size predictor (snout-vent length, SVL), hemipenial truncus length (TL), hemipenial lobes length (LL) and hemipenial total length (HTL). Values are given in millimeters.

| Voucher | SVL | TCL | LL | TTL |
| --- | --- | --- | --- | --- |
| LACV-E-011 | 12.37 | 0.39 | 0.20 | 0.59 |
| LACV-E-053 | 10.11 | 0.26 | 0.25 | 0.51 |
| LACV-E-057 | 11.76 | 0.49 | 0.52 | 1.01 |
| LACV-E-009 | 14.82 | 0.33 | 0.49 | 0.83 |
| LACV-E-060 | 12.29 | 0.27 | 0.70 | 0.98 |
| LACV-E-064 | 13.54 | 0.46 | 0.78 | 1.24 |
| LACV-E-065 | 15.22 | 0.53 | 1.14 | 1.67 |
| LACV-E-067 | 16.12 | 0.63 | 0.95 | 1.58 |
| LACV-E-070 | 13.83 | 0.47 | 1.06 | 1.53 |
| LACV-E-078 | 19.99 | 0.33 | 1.31 | 1.64 |
| LACV-E-080 | 18.43 | 0.34 | 1.23 | 1.57 |
| LACV-E-086 | 17.39 | 0.50 | 1.06 | 1.56 |
| LACV-E-085 | 19.53 | 0.51 | 1.41 | 1.92 |
| LACV-E-084 | 22.77 | 0.66 | 1.71 | 2.37 |
| LACV-E-087 | 22.94 | 0.64 | 1.22 | 1.87 |
| LACV-E-088 | 21.48 | 0.51 | 0.85 | 1.36 |
| LACV-E-095 | 24.59 | 0.47 | 1.18 | 1.64 |
| LACV-E-103 | 30.15 | 0.33 | 1.15 | 1.47 |
| LACV-E-104 | 30.00 | 1.01 | 2.14 | 3.15 |

**S1 Table C (next page). Morphometry of adult hemipenes.** Vouchers and measurements for the analyzed adult male specimens: body size predictor (snout-vent length, SVL), hemipenial truncus length (TCL), hemipenial truncus width (TW), hemipenial lobes length (LL) and hemipenial total length (HTL). Values are given in millimeters.

|  |  | Right Hemipenis | | | |  | Left Hemipenis | | | |
| --- | --- | --- | --- | --- | --- | --- | --- | --- | --- | --- |
| Voucher | SVL | TCL | TW | LL | TTL |  | TCL | TW | LL | TTL |
| LACV3065 | 83.50 | 8.09 | 2.39 | 5.65 | 13.70 |  | 7.58 | 1.86 | 5.79 | 13.37 |
| LACV3078 | 98.50 | 6.91 | 2.09 | 5.89 | 12.80 |  | 7.90 | 2.14 | 6.35 | 14.25 |
| LACV3084 | 112.50 | 7.63 | 3.00 | 8.08 | 15.70 |  | 7.90 | 2.87 | 9.00 | 16.90 |
| LACV3090 | 108.50 | 7.84 | 2.90 | 8.11 | 16.00 |  | 6.80 | 2.35 | 7.62 | 14.42 |
| LACV3092 | 85.50 | 6.45 | 2.28 | 6.34 | 12.80 |  | 6.42 | 1.95 | 6.75 | 13.17 |
| LACV3093 | 89.00 | 5.49 | 2.30 | 6.40 | 11.90 |  | 6.94 | 1.98 | 8.10 | 15.04 |
| LACV3097 | 117.00 | 7.33 | 2.15 | 9.99 | 17.30 |  | 6.45 | 2.41 | 8.18 | 14.63 |
| LACV3098 | 112.00 | 4.40 | 3.29 | 8.66 | 13.10 |  | 7.83 | 2.67 | 9.60 | 17.43 |
| LACV3099 | 96.00 | 5.77 | 2.10 | 6.11 | 14.60 |  | 5.37 | 2.07 | 4.29 | 11.69 |
| LACV3100 | 111.00 | 8.50 | 2.73 | 9.06 | 17.20 |  | 7.40 | 2.49 | 8.91 | 17.25 |
| LACV3114 | 112.00 | 8.16 | 2.77 | 10.38 | 17.20 |  | 8.34 | 2.78 | 10.59 | 17.06 |
| LACV3115 | 97.00 | 6.84 | 2.24 | 5.81 | 13.00 |  | 6.47 | 1.78 | 5.91 | 13.80 |
| LACV3116 | 114.00 | 7.21 | 2.95 | 8.52 | 17.70 |  | 7.89 | 2.60 | 8.32 | 17.18 |
| LACV3117 | 115.00 | 9.17 | 3.25 | 10.27 | 18.40 |  | 8.86 | 2.98 | 10.23 | 19.02 |
| LACV3228 | 107.00 | 8.12 | 2.76 | 9.42 | 17.50 |  | 8.79 | 2.35 | 9.04 | 15.36 |
| LACV3229 | 84.00 | 5.95 | 2.08 | 8.64 | 14.60 |  | 6.32 | 2.35 | 8.42 | 14.64 |
| LACV3269 | 99.00 | 5.72 | 2.36 | 5.86 | 11.60 |  | 6.22 | 2.17 | 7.10 | 12.80 |
| LACV3289 | 107.50 | 7.91 | 2.34 | 9.09 | 17.00 |  | 5.70 | 2.11 | 6.90 | 15.11 |
| LACV3290 | 87.00 | 5.52 | 2.04 | 7.96 | 13.50 |  | 8.21 | 2.47 | 9.32 | 17.53 |
| LACV3291 | 109.00 | 6.95 | 2.59 | 7.19 | 14.10 |  | 8.31 | 2.56 | 7.09 | 15.40 |

| Voucher | SVL | FPUR | FUC | FIGL |
| --- | --- | --- | --- | --- |
| LACV3314 | 82.30 | 5.52 | 4.90 | 10.42 |
| LACV3312 | 90.00 | 4.72 | 4.38 | 9.10 |
| LACV3310 | 82.00 | 2.93 | 4.48 | 7.41 |
| LACV3311 | 91.50 | 3.55 | 4.99 | 8.54 |
| LACV3133 | 79.00 | 5.25 | 5.72 | 10.97 |
| LACV3132 | 75.00 | 4.29 | 5.22 | 9.51 |
| LACV3112 | 101.00 | 4.84 | 4.78 | 9.62 |
| LACV3134 | 96.00 | 4.62 | 5.39 | 10.02 |
| LACV3124 | 101.00 | 6.22 | 7.25 | 13.48 |
| LACV3110 | 91.00 | 5.59 | 5.06 | 10.65 |
| LACV3119 | 88.00 | 7.44 | 6.69 | 14.14 |
| LACV3126 | 97.00 | 5.19 | 4.37 | 9.56 |
| LACV3129 | 93.00 | 4.10 | 4.14 | 8.24 |
| LACV3083 | 99.00 | 4.55 | 4.35 | 8.91 |
| LACV3128 | 90.00 | 6.03 | 5.99 | 12.03 |
| LACV3136 | 90.00 | 3.81 | 4.33 | 8.14 |
| LACV3251 | 88.50 | 4.46 | 4.62 | 9.08 |
| LACV3254 | 84.50 | 4.49 | 5.28 | 9.78 |
| LACV3247 | 104.50 | 5.45 | 6.14 | 11.59 |
| LACV3253 | 91.50 | 3.98 | 6.52 | 10.50 |

**S1 Table D. Morphometry of adult female cloaca.** Vouchers and measurements for the analyzed adult female specimens: body size predictor (SVL), proctodeal-urodeal region length (FPUR), urodeal corns length (FUC), and female internal genitalia length (FIGL). Values are given in millimeters.
